# Multicriteria Decision Model and Thermal Pretreatment of Hotel Food Waste for Robust Output to Biogas: Case Study from City of Jaipur, India

**DOI:** 10.1155/2018/9416249

**Published:** 2018-09-16

**Authors:** Paras Gandhi, Kunwar Paritosh, Nidhi Pareek, Sanjay Mathur, Javier Lizasoain, Andreas Gronauer, Alexander Bauer, Vivekanand Vivekanand

**Affiliations:** ^1^Centre for Energy and Environment, Malaviya National Institute of Technology, Jaipur 302017, Rajasthan, India; ^2^Department of Microbiology, School of Life Sciences, Central University of Rajasthan, Bandarsindri, Kishangarh, Ajmer 305801, Rajasthan, India; ^3^University of Natural Resources and Life Sciences, Vienna, Department of Sustainable Agricultural Systems, Institute of Agricultural Engineering, Konrad-Lorenz-Strasse 24, 3430 Tulln, Austria; ^4^Biogest Energie- und Wassertechnik GmbH, Büropark Donau, Inkustraße 1-7/4/2. OG, 3400 Klosterneuburg, Austria

## Abstract

The anaerobic batch test (45 days at 37°C) was performed to describe the effect of thermal pretreatment at moderate temperatures (60, 80, and 100°C) over durations of 10 and 20 minutes on the enhancement of biogas production using hotel food waste from city of Jaipur, India. The results showed that the total cumulative biogas production with thermal pretreatment (100°C, 10 minutes) was 41% higher than the control. Also, this alternative gets first rank using multicriteria decision making model, VIKOR. This outcome was obtained due to the enhancement of degradation of organic compounds such as protein and volatile solids that occurred in the linear trend. Modified Gompertz and Logistic models were used to study the effect of different pretreatment parameters on lag time and biogas yield. Scanning electron microscopy and Fourier transform infrared spectroscopy were also employed to investigate the effect of thermal pretreatment on the physiochemical properties of food waste.

## 1. Introduction

**Figure 1:**
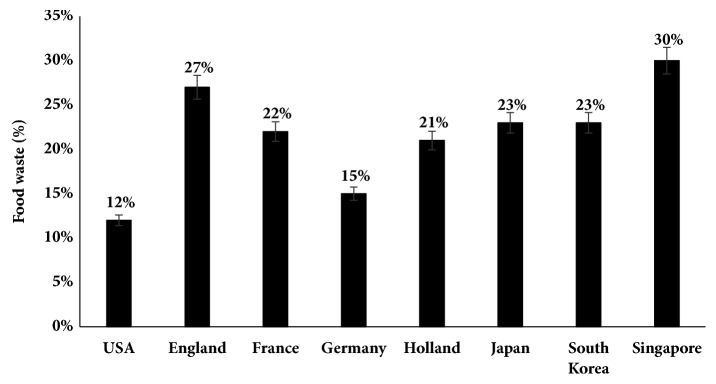
Food waste percentage in different countries [13].

Together with the rapid growth of urban population and changes in the typical eating patterns, management of food waste (FW) has become an issue of international level [1, 2]. In India only, 40% of produced food is lost and wasted, prior to consumption every year [3]. Around the globe, 12-30% FW is generated annually (Figure 1). This huge amount of waste, if either cannot be utilised fruitfully or disposed of carelessly, can hamper public health and generate number of challenges in terms of environmental protection and denotes noteworthy amount of potential energy [1, 2].

In India, the increase in foreign tourist arrivals has pushed the hospitality sector for enhancing quality services in order to fulfil customer's satisfaction. This leads to the growth of hotels as well as FW generation, which has not been explored earlier. Rajasthan, which has a significant share of foreign tourism in the country with 7.2% Foreign Tourist Arrivals (FTA) in 2013 [4], has witnessed rapid growth in hotel infrastructure resulting in a hike of 53.6% in number of hotels available from 2006 to 2016 (Ministry of Tourism, 2016; [5]). This rapid growth calls for another challenge of managing the huge lump of waste and its proper disposal. In most instances, the FW, which consists of rich nutrients capable of providing valuable products and energy, is wasted by being dumped along with municipal solid waste (MSW) due to lack of segregation. Recently, our group has published a comprehensive review regarding the quantification of FW generated from hotels and its energy potential in Jaipur, India (Gandhi et al., 2017).

FW from hotels of Jaipur contains variety of materials, some of which are not suitable for anaerobic digestion (AD), namely, egg shells, coffee grounds, tissue papers, and bone, that have different physical and chemical characteristics. Due to presence of above undesirable components, it is not easy for microbes to degrade these complex and hard materials. Therefore, to improve the biodegradability of these materials and provide readily available organics for maximum biogas recovery, pretreatment becomes necessary prior to AD [6]. Among numerous pretreatment technologies, here more focus is given on thermal pretreatment technology of low severity such as liquid hot water (LHW).

LHW pretreatment is one of the preferred pretreatment methods that can easily enhance the access of sugars to microbes by solubilizing them using higher temperature [7]. Generation of organic acids, such as acetic acid, after hydrolysis, further enhances the rate of conversion of complex sugars to simple sugars [8]. The main advantage of this pretreatment is that it is chemical free and does not need corrosion resistive material for reactors. Kuo and Cheng [9] reported the impact of thermal pretreatment on the AD of kitchen waste at different temperatures (37, 50, and 60°C) to understand the effect on hydrolysis. As a result, pretreatment at 60°C was desirable with a total chemical oxygen demand (TCOD) removal efficiency of 79.2%. Another study from Ariunbaatar et al. [10] revealed that pretreatment at lower temperature (50 and 80°C) for shorter duration, i.e., less than 12 and 1.5 hours, enhances the biogas yield by 40% due to higher solubilization, whereas Ma et al. [11] obtained an increase of 24% in biomethane production at 120°C due to enhanced biodegradation of FW. Li et al. [12] and Jin et al., (2016) investigated the effect of thermal pretreatment not only on kitchen waste at low (55-90°C) and high (120 and 160°C) temperatures on the biogas production, but also on the degradation efficiency of fat, oil and grease, crude protein, volatile solids, volatile fatty acids, etc.

The degradation of fermentable sugars due to high severity of thermal pretreatment can be controlled by maintaining the optimum pH with the addition of base [14]. Thus, to reduce the application of chemicals while mitigating the deterioration of fermentable sugars, milder pretreatment methods should be followed.

The impacts of thermal pretreatment on the chemical and physical properties of three municipal solid wastes (MSW), namely, kitchen waste, vegetable waste, and waste activated sludge, were explored by Liu et al. [15]. Outcomes revealed that thermal pretreatment (175°C, 60 min) diminished viscosity and increased COD, dissolvable sugar, and proteins. In the study, a decrease of 7.9 and 11.7% in the methane yield was observed for kitchen waste and vegetable waste. The authors ascribed this phenomenon and occurrence to the arrangement of an intractable copolymer, melanoidin. Under comparative working conditions (170°C, 1 h), Qiao et al., [16] found that both biogas and methane generation from anaerobically treated waste diminished by 3.4% and 7.5%, respectively. Ma et al. [11] acquired 24% enhancement in biomethane generation with FW pretreated at 120°C.

In spite of the advantages, thermal pretreatment has some disadvantages, i.e., (a) less stability, (b) formation of inhibitors due to side reactions, and (c) higher operating cost [17, 18]. The rate of biochemical reaction controls the hydrolysis and acidogenesis steps, which can be accelerated at high temperatures. The higher amount of fatty acids, ammonia, and so on causes inhibition to methanogenesis process [19].

A series of batch experiments were conducted to measure the biogas potential and to compare the effect of thermal pretreatment using the hotel FW as the substrate. Both the increased hydrolysis and improved solubilization depends on temperature and time [20]. In the present study, the batch AD of pretreated and untreated hotel FW was performed to derive the critical parameters, namely, temperature and time affecting biogas production, and to relate them to the impact of severity on the chemistry of substrate. Physical and chemical fingerprinting was performed for the untreated and pretreated substrate using SEM and FTIR, respectively. Energy analysis was also performed using the data generated to study the economics of the pretreatment process.

## 2. Material and Methods

### 2.1. Food Waste and Inoculum Characteristics

FW used in this study was collected on the weekly basis from the cafeteria and kitchens of the six hotels reputed with star category in Jaipur. After manual sorting, FW was separated from undesirable parts (such as egg shells, bones, tissue papers, peels, and kernels). Collected FW mainly consisted of carbohydrates, proteins, and fats, being the major components of rice, vegetables and fruits, bread, cooked pulses, and meat. All parts of collected food were mixed in a kitchen blender and stored at 4°C in a refrigerator after crushing into particles with an average size of 1-2 mm.  Table 1 shows the basic characteristics of FW and inoculum used in this experiment.

Inoculum was taken from the anaerobic digester plant (Rajasthan Gau Sewa Sangh, Durgapura, Jaipur, Rajasthan), which was using cow manure at the mesophilic temperature range, with initial pH of 7.8. Volatile solid (VS) concentration of the inoculum was 68.75% VS/TS. Before the experiment, it was stored in a container at room temperature for five days to acclimatize and starve it prior to using in AD batch experiments.

### 2.2. Thermal Pretreatment

Thermal pretreatment was performed using hot water bath (Sanco, India) and 50g of a representative sample of FW (wet mass) and 50ml of distilled water to provide uniform heating for pretreatment making the solid to liquid ratio of 1:1 (w/v). The water bath was kept on until the target temperature reached to 60, 80, and 100°C in the beaker and held for the selected durations of time (10 and 20 min) for each temperature (Table 2). During the pretreatment process, the mixture was stirred continuously until the set period completed. After the different pretreatments were carried out, the samples were kept in the refrigerator at 4°C till further use. The liquid part was separated from the insoluble remains for further analysis.

To determine how the combined effect of temperature (T) and time (t) affects FW [21], severity factor (Table 2) was calculated using the following equation:

(1) $$\begin{array}{c} \log R_{0}\left\{\;R_{0}\;\right\}=t*\exp\left(\;\frac{T-100}{w}\;\right) \end{array}$$

where t is pretreatment time, T is pretreatment temperature, and w = 14.75 is an empirical parameter.

### 2.3. Anaerobic Digestion and Biogas Production

The FW digestion experiment was performed in the 610 mL serum bottle with active volume of 400mL (37±1°C, 90 rpm, 45 days) using orbital shaker (REMI CIS 24, India) to govern the biodegradability of untreated and thermally treated feedstock. Each bottle was fed with a mixture of FW and inoculum (with 1.2% TS after dilution) corresponding to the final concentration of 1.5g VS L^−1^ with initial pH between 7 and 7.3. Then, the upper space of each bottle was purged with nitrogen for at least 2 min and sealed by rubber plugs to guarantee anaerobic conditions. For each sample, the experiment was run in triplicate. Simultaneously, bottle containing inoculum alone was also made to measure the biogas generated from the inoculum. Initially the pressure (millibars) accumulated in bottle was measured by a pressure meter at the following time points: days 1, 2, 3, 4, 6, 9, 12, 15, 18, 22, 27, 32, and 45, which was further used to calculate the volume of biogas produced in each bottle using the following ideal gas law equation:

(2) $$\begin{array}{c} V_{biogas}=\frac{\left(P.V_{head.}C\right)}{R.T} \end{array}$$

whereV_Biogas_ is daily biogas volume (L),P is absolute pressure difference (mbar),V_Head_ is volume of the head space (L),C is molar volume (22.41 mol^−1^), R is universal gas constant (83.14 L mbar K^−1^ mol^−1^),T is absolute temperature (K).

 Biogas yield was calculated by taking the average of the biogas produced per VS of added substrate in triplicate bottles. After the pressure measurement, each bottle was depressurized by penetrating the needle into the rubber cap. The reported experimental results demonstrate the mean of triplicate made for each sample. Reported biogas yields from the substrates were calculated by subtracting the biogas production of the inoculum from the gross biogas production of the substrates.

### 2.4. Data Fitting Using Models

To assess the performance parameter two models were used. The Modified Gompertz Equation (GM) (3) was used to analyse and describe the biogas production. This model was based on the direct relation between the biogas production and microbial activity during the occurrence of different phases of AD [15, 22].

(3) $$\begin{array}{c} CBP=B\exp\left\{\;-\exp\left[\;R_{b}e\frac{\alpha -t}{B}+1\;\right]\;\right\} \end{array}$$

where CBP is the cumulative biogas production (mL/g VS) at digestion time t days; B is biogas potential maximum production (mL/g VS); R_b_ is maximum biogas production rate (mL/g VS); e is exp⁡(1) = 2.718; ‘*α*' is duration of lag phase (d).

Logistic function (LF) depends on the initial exponential increase that fits the global biogas production. This model assumes that the rate of biogas production is proportionate to the volume of gas already produced, the maximum production rate, and the maximum capacity of biogas production [23].

(4) $$\begin{array}{c} CBP=\frac{B}{\left(1+\exp\left[4R_{b}\left(\alpha -t\right)/B+2\right]\right)} \end{array}$$

Equation terms have same meaning as explained in (3).

Lag phase (*α*) is an important factor indicating the efficiency of biogas production, which was calculated in both the above equations [24, 25]. The kinetic parameters of each of the batch bioreactors were estimated using nonlinear least square regression analysis (see (3) and (4)).

### 2.5. Energy Analysis

Energy content in the generated biogas was calculated on the basis of methane content available in the gas (average, 62%), which was calculated using the ultimate analysis (Table 1) and Boyle's equation (5) [26]:

(5) CcHhOoNnSs+144c−h−2o+3n+2sH2O⟶184c−h+2o+3n+2sCO2+184c+h−2o−3n−2sCH4+nNH3+sH2S

Then, initial VS based biogas volume (mL) was multiplied by the lower heating value of methane (33 kJ/L at 25°C and 1.01 bar). Heating load needed to keep the digester at particular temperature was not calculated because, during the practical application such as combined heat and power (CHP) systems, the system itself can typically meet the required load [20]. For the calculation of energy needed to heat the FW during pretreatment, (6) was used [20]:

(6) Energy  input  for  material  heating  KJ/kg  initial  VS= =mw∗Cw∗Δt

where m_w_ is the mass of water required in kg per kg of VS of FW sample (367 kg/kg initial VS), c_w_ is the specific heat of water (4.2 kJ/kg/°C), and Δt is the increase in temperature (°C, from 30°C to the required temperature).

Gain/loss in the biogas energy (kJ/kg initial VS) was then measured by subtracting the biogas energy from the energy given for the hot water pretreatment.

### 2.6. Analytical Methods

The TS and VS content of FW was determined according to the standard methods [27]. The pH value was measured by a pH meter (LMPH 10, Labman Scientific Instruments Pvt. Ltd., India). TCOD was measured by using the closed reflux method [28]. SCOD was analysed according to Finnish standard SFS 5504 [29]. The ultimate analysis was performed using the instrument model FLASH EA 1112 series (Thermo Finnigan, Italy). The soluble protein content was estimated by Lowry's method [30]. The Scanning electron microscopy (SEM) (Nova NanoSEM 450, Netherland) analysis was done to observe the microstructure and morphological difference between the untreated and treated samples of pulverised FW samples. Liquid samples were mounted on an aluminium stubs (10mm diameter) using double coated tape. A Fourier transform infrared (FTIR) spectrometer of spectrum 10.4.00 (PerkinElmer, USA) was employed to examine the chemical composition of untreated and treated FW samples.

### 2.7. Multicriteria Decision Making Model

Multicriteria decision making models (MCDM) are normally applied for both indefinite set of scenarios and definite set of scenarios. Pretreatment of FW is a definite set of scenarios having a definite set of output. For definite set of scenarios, there are many MCDM techniques such as ELECTRE (elimination et choix traduisant la realité), PROMETHEE (preference ranking organization method of enrichment evaluation), TOPSIS (technique for order preference by similarity to ideal solution), and VIKOR (VlseKriterijuska Optimizacija I Komoromisno Resenje) [31]. Several previous studies have applied VIKOR technique in the field of renewable and sustainable energy [32]. The steps involved in VIKOR method are described below.

**Step 1 .**

Create a decision matrix of alternative selected for experiment and output.

(7) $$\begin{array}{c} D_{mn}=\left[\begin{array}{ccc} x_{11} & x_{12} & x_{1m}\\ x_{21} & \ldots & \ldots \\ x_{n1} & \ldots & \ldots \end{array}\right] \end{array}$$

**Step 2 .**

Create a normalized matrix using

(8) $$\begin{array}{c} \rho_{ij}=\frac{x_{ij}}{\sum_{i=1}^{n}x_{ij}} \end{array}$$

**Step 3 .**

After creating normalized matrix, find entropy of each alternative

(9) $$\begin{array}{c} E_{j}=-k\overset{m}{\underset{i=1}{\sum }}\rho_{ij}\ln\left(\;\rho_{ij}\;\right) \end{array}$$

where k = 1/ ln (m)

**Step 4 .**

Calculate dispersion value of each alternative

(10) $$\begin{array}{c} \pi_{j}=1-E_{j} \end{array}$$

**Step 5 .**

Find weight of each alternative

(11) $$\begin{array}{c} \omega_{j}=\frac{\pi_{j}}{\sum_{j=1}^{n}\pi_{j}} \end{array}$$

**Step 6 .**

Determine utility measure (*α*_*i*_) and regret measure (*β*_*i*_) using weights of each alternative

(12) αi=∑j=1nωjxijmax−xijxijmax−xijminif  j  is  benefit  criteria  for  j=1,2…m

(13) αi=∑j=1nωjxij−xijminxijmax−xijminif  j  is  cost  criteria  for  j=1,2…m,

(14) βi=max⁡ofωjxij−xijminxijmax−xijminfor  j=1,2,…m

from decision matrix; obtain maximum (*x*_*ijmax*_) and minimum (*x*_*ijmin*_) value for each output.

**Step 7 .**

Finally calculate VIKOR index, *Ω*_*i*_

(15) $$\begin{array}{c} \Omega_{i}=\varepsilon \left(\;\frac{\alpha_{i}-\alpha_{i}^{-}}{\alpha_{i}^{+}-\alpha_{i}^{-}}\;\right)+\left(\;1-\varepsilon \;\right)\left(\;\frac{\beta_{i}-\beta_{i}^{-}}{\beta_{i}^{+}-\beta_{i}^{-}}\;\right) \end{array}$$

where  *α*_*i*_^+^  *and*  *β*_*i*_^+^ = max  ⁡*of*  *α*_*i*_  *and*  *β*  (*i* = 1,2,…..*m*) and *α*_*i*_^−^   *and*  *β*_*i*_^−^ = min⁡  *of*   *α*_*i*_   *and*   *β*  (*i* = 1,2,…..*m*) .

*ε* is introduced as weight for the maximum value of utility and (1 − *ε*) is the weight of the individual regret and normally its value of *ε* is taken as 0.5.

### 2.8. Statistical Analysis

All the data were tested for the level of significance and analysis of variance (ANOVA; p<0.05) was performed in Microsoft excel spreadsheet (version 2016) using solver function.

## 3. Results and Discussion

### 3.1. Effects of Thermal Pretreatment on Degradation of FW

#### 3.1.1. pH

**Figure 2:**
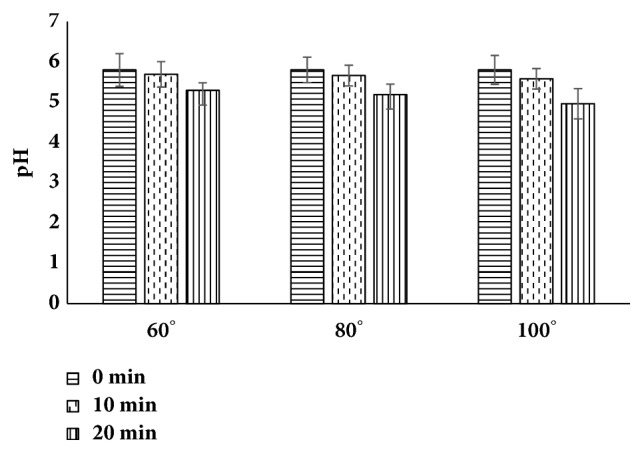
Variation in pH after thermal pretreatment of hotel food waste (two-factor ANOVA of data set showed p = 0.001662).

Figure 2 shows the effect of pretreatment temperature and duration time on the pH of FW. The untreated sample was mild acidic in nature (pH 5.8). A significant decrement in the pH was observed following the thermal pretreatment of FW. The increase in pretreatment temperature from 60° to 100°C led to drop in pH. The duration of pretreatment further decreased the pH (Figure 2). There is strong dependence of pH decrease on severity factor values (R^2^ = 0.98 in quadratic regression and 0.71 in linear regression) also (Table 2) that may be due to release of the sugars followed by pretreatment.

Thus, progressive increase in pretreatment severity resulted into decrease in pH, which may be due to the phase change of organic acids from solid to liquid in the FW. Other reasons could be the thermal hydrolysis reaction, i.e., high-pressure boiling of waste followed by rapid decompression, which leads to the reduction in pH. This process increases the biodegradability of waste along with reduction in the microbial load to an extent depending on temperatures and time.

#### 3.1.2. VS

**Figure 3:**
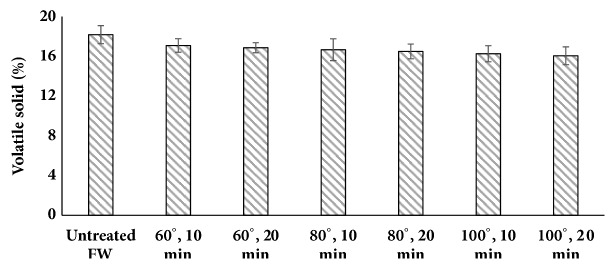
Volatile solid proportion after thermal pretreatment in hotel food waste (two-factor ANOVA of data set showed p = 0.000025).

The VS content for the untreated FW was 18.18 ± 1.33%. It can be concluded that, as pretreatment severity increases, no notable decrease in the VS content of the substrates was observed (Figure 3). The negligible decrease in VS may be attributed to the high temperatures entailing the loss of volatile substances and thus the decline in final VS content [33].

#### 3.1.3. SCOD

**Figure 4:**
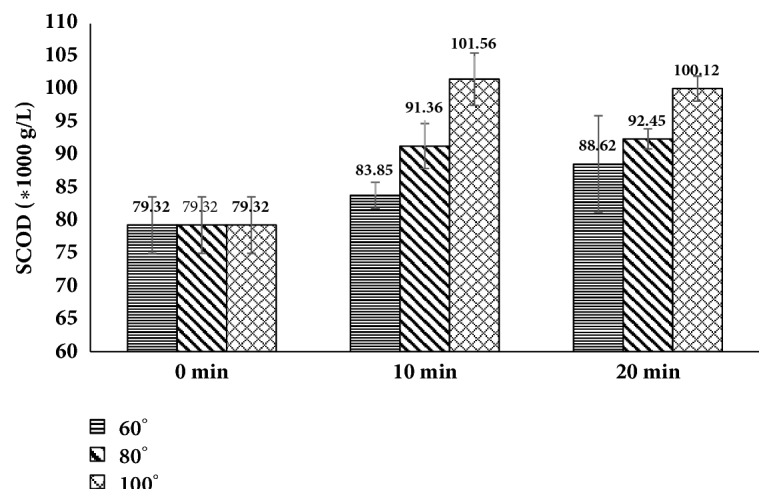
Effect of pretreatment on SCOD content of hotel food waste (two-factor ANOVA of data set showed p = 0.03).

It is a parameter, which can be used as a performance indicator of digestion process in AD. It reflects the amount of soluble organic matter present in the substrates in the form of dissolved organic matter.  Figure 4 depicts SCOD for untreated and pretreated FW. For pretreatment temperatures (60, 80, and 100°C), the observed increase in the SCOD content was marginally significant and the values were about to get stable after 10 min. The maximum solubilization was observed at 100°C, 10 min which was around 28%, while it was around 16 and 11% for pretreatments performed at 80 and 60°C for 20 min, respectively.

The obvious reason for the increased SCOD content on increasing the pretreatment temperature and duration is the breakage of chemical bonds, including VFA's, polysaccharides, and proteins, by providing external energy in form of heat [33].

The results above support the liquid hot water pretreatment as the preferred choice for enhancing the digestion of FW. Kondusamy et al. [34] also observed the improvement in biogas generation from liquid hot water pretreated FW. Furthermore, it may also increase the production of digestate/slurry due to the transformation of organics in FW from solid to liquid.

#### 3.1.4. Soluble Protein

**Figure 5:**
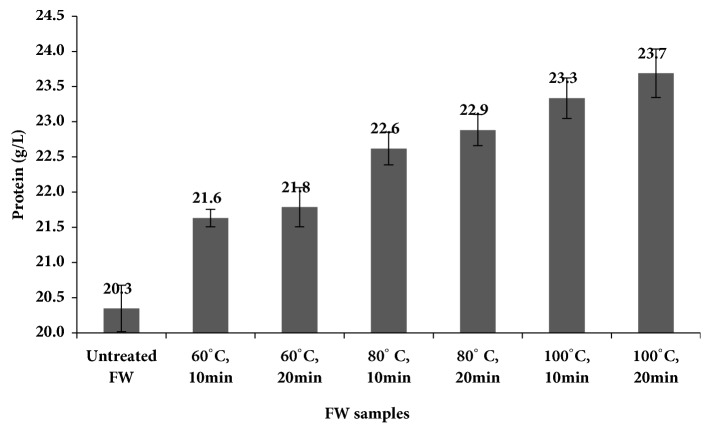
Protein solubilization in hotel food waste (two-factor ANOVA of data set showed p = 0.0000009).

Figure 5 illustrates the solubilization of FW protein after pretreatment. The pretreatment was observed to affect the protein content of FW in a similar trend as the COD solubilization. The protein solubilization increased linearly with temperature (Figure 5). The highest protein solubilization of around 16% was obtained at pretreatment condition of 100°C and 20 min.

#### 3.1.5. Effects of Pretreatment on FW Functional Groups

**Figure 6:**
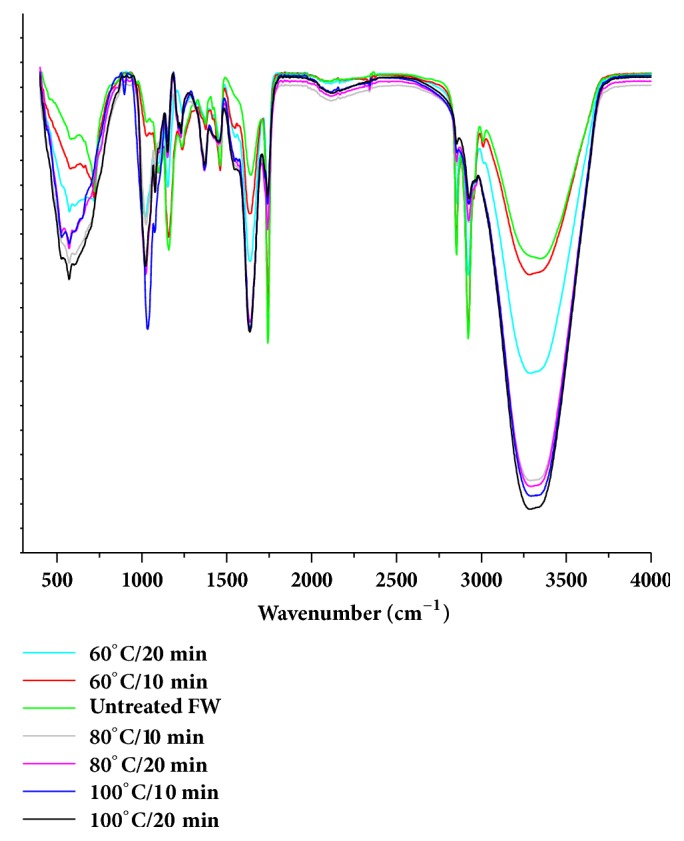
FTIR spectra of thermally pretreated hotel food waste.

FT-IR analysis was performed in the range of 4000-400 cm^−1^ to characterise the effect of pretreatment on the functional groups and the chemical structure of untreated and pretreated FW (Figure 6). The peak within the range of 3000 to 3600 cm^−1^ showed the combined presence of –OH group from the internal water and N-H group from the amide I of FW. The peak intensity was observed to increase with pretreatment severity because of solubilization of complex insoluble organics to simpler soluble organics. However, above 80°C, the increment in the –OH stretching vibrations did not remain significant. At wavelengths 1655 and 1545 cm^−1^, peaks of C=O and C-N stretching vibrations of amide I and II of protein were observed [35, 36]. Since degradation of protein occurred during the pretreatment, peaks of amino acids or smaller fragments of carboxyl group and NH_3_ become wider with temperature and duration. No significant change was detected in the other functional groups for the analysed samples.

#### 3.1.6. Scanning Electron Microscopy (SEM)

**Figure 7:**
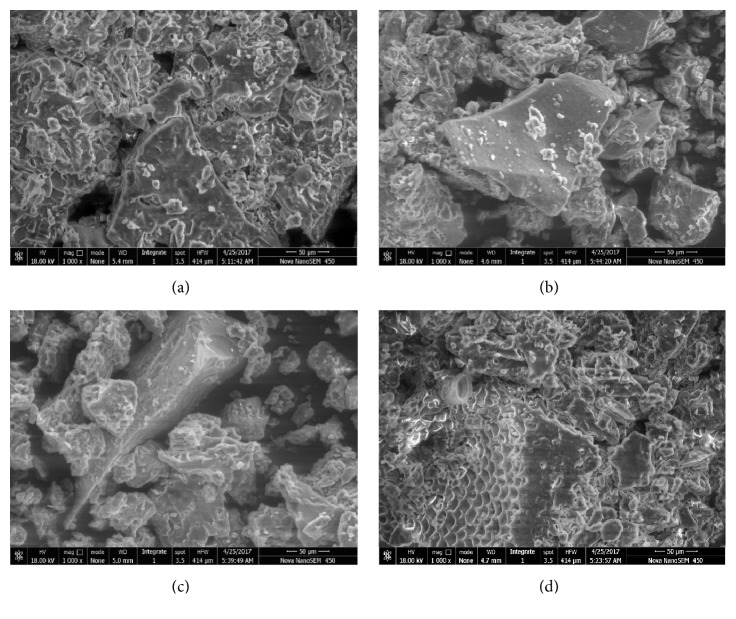
SEM images of FW under magnification of 1000x. (a) Untreated FW, (b) 60°, 10min, (c) 80°, 10min, and (d) 100°, 10min.

SEM assists to understand the effect of thermal pretreatment on the microstructural properties of FW. Clusters of varying sizes and shapes were observed in the scanning electron micrographs, which were the composites of carbohydrates, proteins, and lipids (main organic compounds in FW). In contrast to treated FW, untreated FW contains more compact and cemented particles, which possess flat, rigid, and smooth surfaces of size ranging between 30 and 50 *µ*m (Figure 7(a)). Figures 7(b), 7(c), and 7(d) illustrated the disruption of bigger particles into smaller rugged, rough, and serrated surface particles of sizes 10-30 *µ*m following pretreatment.

Therefore, it can be concluded that liquid hot water pretreatment resulted into decrystallisation of FW that would, in turn, be helpful in enhancing the contact between substrates and microorganisms via enhancing the available surface area for increased biogas production.

### 3.2. Biogas Yield and Production Rate

**Figure 8:**
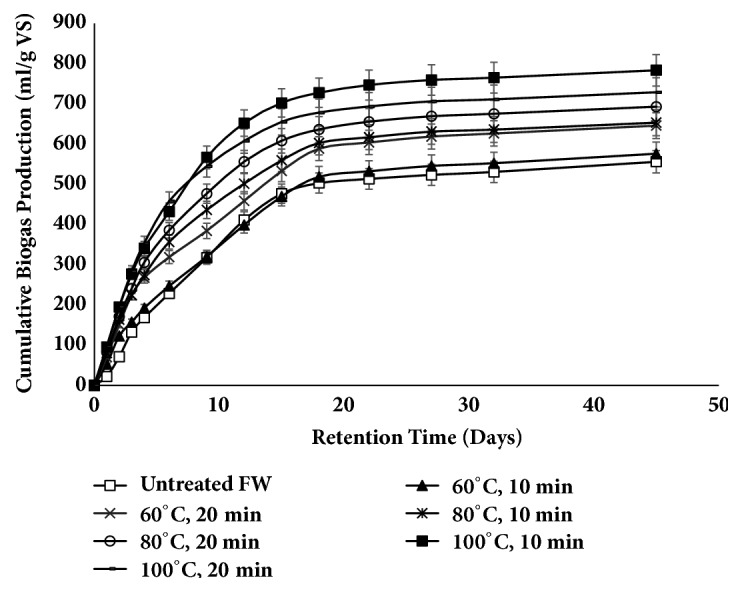
Cumulative biogas production from untreated and thermally pretreated hotel food waste (two-factor ANOVA of data set showed p = 1.00647E-29).

The improvement of cumulative biogas production from untreated and thermally pretreated FW is shown in Figure 8. As the experiment was executed, the biogas production started instantly on the first day of digestion, and primary lag phase was barely observed in pretreated samples, in contrast to untreated FW, which may be due to the less solublised organic content of FW. The production of biogas from FW was significantly increased by the thermal pretreatment under all conditions. The maximum biogas yield of 783 mL/g VS was attained by the treatment temperature at 100°C, 10 min. It showed the impact of moderate temperature pretreatment on augmenting the degradation of FW which was supported by the SCOD findings. Similar biogas production patterns were reported by Li et al. [24]. The negligible lag phase can be identified based on the rapid hydrolysis of solubilized compounds and quick adaptability of the microbes for the substrate [25].

The other curves at temperatures 60° and 80°C with treatment durations 10 and 20 min, respectively, demonstrated analogous trend due to linear increase in the SCOD and protein content, though the biogas production observed was less than the yield attained at 100°C, 10 min.

The development of refractory inhibitory compounds such as melanoidin and increase in concentration of soluble phenol at higher thermal pretreatment temperature and duration (100°C, 20 min) may reduce the activity of methanogens but not utterly after witnessing the high biogas yield compared to untreated FW [33].

The whole process of AD indicated the rapid degradation of feedstock and rather intensive biogas production [24, 37]. Readily available soluble organic compounds in untreated or pretreated FW resulted in rapid biogas production in initial phase of AD. Thus, higher severity was considered as the sole reason to provide higher digestible organics in pretreated FW, compared to the moderately degradable compounds in untreated food waste. The reason behind the sudden decrease in the production rate after the first week is the accretion of VFA's, due to the rapid growth of acidogens in comparison to methanogens, which timely consume VFA's to produce biogas. In turn acidogens increase, leading to reduction in pH out of the optimum range for AD.

Thus, the thermal pretreatment highlights the rapid solubilization of organic and inorganic macromolecules. Moreover, organic compounds with low molecular weight and increased surface area promote the contact between the microbes and substrate [38], resulting in increased conversion of organic materials to biogas.

### 3.3. Kinetic Study

The expected kinetic parameters based on Gompertz and Logistic functions models are shown in Table 3. Parameters such as estimated biogas yield and lag phase duration were also calculated using the equations of the two models. For the validation purpose, the trend lines for experimental cumulative biogas values were plotted against the expected values till 12^th^ day, as the biogas production curve remained steep.

These two models (Gompertz and Logistic model) were also used by group of researchers ([25, 39]; Pariosh et al., 2017) to fit the experimental biogas production curve against the predictions. The relation between the two curves was evaluated on the basis of adjusted R-square and Root mean square error (RMSE).

Biogas production potential of the untreated and treated FW at temperatures 60°, 80° and 100°C (each for 10, 20 min) were measured to be 555, 575, 645, 653, 692, 783 and 728 mL/g VS. Based on final CH_4_ content (average 62%) the methane content were 344, 357, 400, 405, 429, 485 and 451 mL/g VS respectively. The maximum biogas yield was obtained with the pretreatment temperature and duration of 100°C, 10 min. Lag time, RMSE and R^2^ values for both the models, at each operating temperature, are shown in Table 3.

Calculated lag time for both the kinetic models was found to be nearly zero for every case because of the presence of active bacteria in the added inoculum and readily accessible biodegradable component in the FW. It also signifies the rapid consumption of soluble material by anaerobic biomass [25]. The R^2^ values for Gompertz and Logistic model are quite similar (as shown in Table 3) and fall within the range of 0.98-0.99 that ensures the fitting of predicted values to the experimental values. Whereas, The RMSE (%) values for Gompertz model are lower than that of logistic model (between 2-6%), which favours the former model more than the later for the kinetic study of biogas production.

### 3.4. Net Energy Production Analysis

Untreated FW can generate 12.89 MJ/kg (VS basis) of energy via AD. It was expected that pretreatment can improve the net benefit by increasing the biogas yield. However, pretreatment can cause dry matter loss and requires additional energy, which can offset the improvement in biogas yield. As shown in Table 4, the energy outputs of LHW pretreated FW (13.35-18.16 MJ/kg initial VS) are greater than that of untreated FW (12.89 MJ/kg initial VS) due to the enhancement of the solubilization of organic compounds during LHW pretreatment. Moreover, LHW pretreatment requires (46.05-107.48) MJ/kg initial VS for heating up the feedstock, resulting in negative net biogas energy (Table 4). Therefore, LHW pretreatment under the studied conditions is looking less promising but it is suggested that the residual heat of the CHP may be used to pretreat the FW and then followed by biogas production.

### 3.5. Multicriteria Decision Making Modelling

After the analysis was perfomed and lots of data were genrated; for multicriteria analysis of thermal pretreatment of FW, a set of output data was selected for employing VIKOR method. A set of possible alternatives based on number of tretament temperature and time duration was prepared for getting best tretament condition (Table 5). Six different alternatives such as 60, 80 and 100°C pretreatment for 10 and 20 minutes were arranged in a matrix against effect on pH, VS, SCOD, soluble protein and biogas yield after thermal pretreatment of FW.

In Table 5, each set of data have different unit. Making each data dimensionless is prior requirement before applying MCDM methodological approach and making the data uniform for comparison. To make each output dimensionless, normalized matrix was created using equation (8) (Table 6).

After making each entry of the data set dimensionless, entropy value, dispersion value and weight of each alternative was determined (Table 7) using equations (9), (10) and (11).

After getting the values of entropy, dispersion and weight, utility measures, regret measure and VIKOR index were calculated (Table 8) using equations (11), (13), (14) and (15).

Finally, rank of alternatives were provided as per VIKOR index obtained in ascending order, and best alternative is one having minimum value of VIKOR index (Table 9).

The obtained rank showed the effect of pretreatment and time duration on various factors tabulated in Table 5. Alternatives A_5_ and A_6_ obtined first and second best rank for thermal pretreatment of FW. However, time duration may play a vital role despite having same temperature for pretreatment, alternative A_6_ got second rank because of pretreatment time (20 minutes). Alternative A_1_, A_2_, A_3_ and A_4_ secured sixth, fifth, fourth and third rank respectively as per VIKOR approach. This clearly showed that as temperature is reduced for pretreatment, overall performance of reactor is also decreases. Further, the experimental results were in agreement with the rank provided by the VIKOR MCDM approach.

According to MCDM using VIKOR technique, pretreatment of FW at 100°C, 10 min was observed to be the best treatment condition among others and also experimentally this alternative helps to achieve maximum biogas yield (783 ml/g VS).

## 4. Conclusions

Thermal energy was employed for FW pretreatment concerning augmentation of biogas yield in the AD process. A significant enhancement of organic matter solubilization and biogas production from FW was observed after thermal pretreatment. A direct correlation between soluble COD, soluble protein and biogas production was observed. However, the soluble COD did not increase after the temperature and duration of (100°C and 10 min). Thermal pretreatment enhances the degradation of organic and inorganic compounds, which leads to efficient AD of FW treated at high temperature and longer duration. SEM analysis showed that the thermal pretreatment disrupts and reduces the size of the food particles and increases the roughness to promote the contact between the substrate and microbes. Whereas, FTIR also showed the presence of carbohydrates, proteins and lipids as well as their conversion in to simpler forms with the increasing severity of pretreatment. Both kinetics models (Modified Gompertz and Logistic function) showed agreement with the experimental curve and fit up to the similar extent with R^2^ value greater than 95%. Net energy analysis showed that thermal pretreatment was not an economical method of pretreatment as it incurs more energy as input than additional output energy from biogas. Therefore, in the present form it may have less feasibility; however employment of residual CHP heat for FW pretreatment may lead to development of an energy efficient, sustainable, and economic process.

## Figures and Tables

**Table 1:** Analysis of the FW and inoculum.

**Parameters**	**F** **W** ^**a**^	**Inoculum**
*Proximate analysis*		
pH	5.1 ± 0.2	7.8
Total solids (%)	35.8 ± 0.5	6.4 ± 0.2
VS (%, wet basis)	34.3 ± 0.4	4.4 ± 0.8
TCOD (mg/L)	229.5 ± 38.7	-
SCOD (mg/L)	79.3 ± 4.3	-
Protein (mg/L)	20.3 ± 0.3	-
*Ultimate analysis*		
Carbon (%)	49.5 ± 1.6	35.1 ± 1.2
Hydrogen (%)	9.9 ± 0.2	4.3 ± 0.4
Nitrogen (%)	2.7 ± 0.4	1.7 ± 0.2
Oxygen (%)	36.1 ± 1.7	58.8 ± 0.9
Sulphur (%)	0.3 ± 0.0042	-
C/N	18.33	20.64

^a^Each parameter was measured in triplicates: SCOD, soluble COD; TCOD, total COD.

**Table 2:** Severity factor and pretreatment conditions of hotel food waste.

**Sl. No.**	**Severity factor**	**Pretreatment conditions**
1	0.0	Untreated
2	1.7	60°C, 10 min
3	2.0	60°C, 20 min
4	2.3	80°C, 10 min
5	2.6	80°C, 20 min
6	2.9	100°C, 10 min
7	3.2	100°C, 20 min

**Table 3:** Results of kinetic study-modified Gompertz model and Logistic model.

**Parameter**	**Untreated FW**	60°**C, 10min**	60°**C, 20min**	80°**C, 10min**	80°**C, 20min**	100°**C, 10min**	100°**C, 20min**
Cumulative biogas production-experimental*∗*	410	398	458	501	556	652	607
**Modified Gompertz**							
Cumulative biogas production-predicted*∗*	410.31	396.98	452.10	482.45	541.98	627.64	553.22
Lag phase (days)	0.5	0.06	0.05	0.03	0.02	0.01	0
R^2^	0.9909	0.9666	0.9528	0.9733	0.9837	0.978	0.9837
RMSE (%)	5.95	2.95	2.07	2.34	2.56	2.02	1.97
**Logistic Model**							
Cumulative biogas production-predicted*∗*	410.93	398.49	457.04	494.61	551.86	643.16	577.48
Lag phase (days)	0	0	0	0	0	0	0
R^2^	0.9937	0.9698	0.9573	0.9757	0.9875	0.9814	0.9852
RMSE (%)	6.73	14.1	15.54	12.42	9.58	11.25	10.8

*∗*Value was taken until 12^th^ day.

**Table 4:** Net benefit of energy production from biogas due to the thermal pretreatment of FW.

**Pretreatment conditions**	**Severity factor**	**Total biomass energy production** **(MJ/kg VS)**	**Energy given for the pretreatment** **(MJ/kg VS)**	**Net biogas energy production** **(MJ/kg VS)**
60°, 10min	1.7	13.35	46.06	Negative
80°, 10min	2.3	15.15	76.66	Negative
100°, 10min	2.9	18.16	107.46	Negative
Untreated FW	0	12.89	0	12.89

**Table 5:** Decision matrix as per experimental results of thermal pretreatment of FW.

**Alternatives**	**pH (C**_**1**_ **)**	**VS reduction (**%**) (C**_**2**_**)**	**sCOD** **(g/L) (C**_**3**_**)**	**Soluble protein (g/L) (C**_**4**_ **)**	**Biogas yield (mL/g VS) (C**_**5**_ **)**
60°C, 10 minutes (A_1_)	5.69	17.09	83.85	21.6	575.66
60°C, 20 minutes (A_2_)	5.29	16.87	88.62	21.8	645.73
80°C, 10 minutes (A_3_)	5.66	16.66	91.36	22.6	653.24
80°C, 20 minutes (A_4_)	5.19	16.49	92.45	22.9	692.68
100°C, 10 minutes (A_5_)	5.58	16.26	101.56	23.3	783.23
100°C, 20 minutes (A_6_)	4.96	16.09	100.12	23.7	728.48
Sum	32.37	99.46	557.96	135.9	4079.02
Max	5.69	17.09	101.56	23.7	783.23
Min	4.96	16.09	83.85	21.6	575.66
Max–min	0.73	1	17.71	2.1	207.57

**Table 6:** Normalized matrix is obtained as below for each alternative, criterion using (8).

	**C**_**1**_	**C**_**2**_	**C**_**3**_	**C**_**4**_	**C**_**5**_
A_1_	0.175780043	0.171827871	0.15027959	0.158940397	0.141127035
A_2_	0.163422922	0.169615926	0.15882859	0.160412068	0.158305181
A_3_	0.174853259	0.167504524	0.163739336	0.166298749	0.16014631
A_4_	0.160333642	0.165795295	0.165692881	0.168506255	0.169815299
A_5_	0.172381835	0.163482807	0.182020217	0.171449595	0.192014258
A_6_	0.153228298	0.161773577	0.179439386	0.174392936	0.178591917

**Table 7:** Determining the value of entropy, dispersion, and weight of each alternative using (9), (10), and (11).

**Alternatives**	**Entropy, ** **E**_**j**_	**Dispersion, ** ***π***_**j**_	**Weight, ** **ω**_**j**_
A_1_	0.999302322	0.1842	0.18328732
A_2_	0.999302322	0.177041	0.176163955
A_3_	0.999302322	0.167215	0.166386118
A_4_	0.999302322	0.168169	0.167336036
A_5_	0.999302322	0.146966	0.146238199
A_6_	0.999302322	0.161388	0.160588372

**Table 8:** Utility measure (*α*_*i*_), regret measure (*β*_*i*_), and VIKOR index (Ω_*i*_) for each alternative and criterion using (12), (13), (14), and (15).

	**C**_**1**_	**C**_**2**_	**C**_**3**_	**C**_**4**_	**C**_**5**_	**α**_**i**_	**β**_**i**_	Ω_**i**_
A_1_	0	0	0.248051576	0.062278006	0.525738008	0.83606759	0.525738008	1
A_2_	0.076771049	0.005241554	0.181241524	0.056346768	0.348263122	0.667864016	0.348263122	0.716785979
A_3_	0.005757829	0.010244856	0.142864262	0.032621813	0.329241623	0.520730382	0.329241623	0.604021944
A_4_	0.095963811	0.014295148	0.127597394	0.023724955	0.229347096	0.490928403	0.229347096	0.486020253
A_5_	0.021112038	0.019774954	0	0.011862477	0	0.05274947	0.021112038	0
A_6_	0.140107164	0.023825246	0.020169072	0	0.138672043	0.322773525	0.140107164	0.290263409

**Table 9:** Rank of alternatives.

**Alternatives**	**VIKOR index**	**Rank**
60°C, 10 minutes (A_1_)	1	6
60°C, 20 minutes (A_2_)	0.716785979	5
80°C, 10 minutes (A_3_)	0.604021944	4
80°C, 20 minutes (A_4_)	0.486020253	3
100°C, 10 minutes (A_5_)	0	1
100°C, 20 minutes (A_6_)	0.290263409	2

## References

[1] Paritosh K., Kushwaha S. K., Yadav M., Pareek N., Chawade A., Vivekanand V. (2017). Food Waste to Energy: An Overview of Sustainable Approaches for Food Waste Management and Nutrient Recycling. *BioMed Research International*.

[2] Paritosh K., Mathur S., Pareek N., Vivekanand V. (2018). Feasibility study of waste (d) potential: co-digestion of organic wastes, synergistic effect and kinetics of biogas production. *International Journal of Environmental Science and Technology*.

[3] Makhija A. (2015). Control Food Wastage, Reduce Malnutrition. *Multidisciplinary Scientific Reviewer*.

[4] India Tourism Statistics at a glance Ministry of Tourism, Government of India. http://ficci.in/spdocument/20610/Report-Tourism-Infrastructure.pdf.

[5] Chedwal R., Mathur J., Agarwal G. D., Dhaka S. (2015). Energy saving potential through Energy Conservation Building Code and advance energy efficiency measures in hotel buildings of Jaipur City, India. *Energy and Buildings*.

[6] Ramzan N., Naveed S., Latif N., Saleemi A. R. (2010). Characterization of Kitchen waste as a Feedstock for Biogas Generation by Thermophilic Anaerobic. *Digestion.-NUST Journal of Engineering and Sciences*.

[7] Di Girolamo G., Bertin L., Capecchi L., Ciavatta C., Barbanti L. (2014). Mild alkaline pre-treatments loosen fibre structure enhancing methane production from biomass crops and residues. *Biomass & Bioenergy*.

[8] Wan C., Li Y. (2011). Effect of hot water extraction and liquid hot water pretreatment on the fungal degradation of biomass feedstocks. *Bioresource Technology*.

[9] Kuo W., Cheng K. (2007). Use of respirometer in evaluation of process and toxicity of thermophilic anaerobic digestion for treating kitchen waste. *Bioresource Technology*.

[10] Ariunbaatar J., Panico A., Yeh D. H., Pirozzi F., Lens P. N. L., Esposito G. (2015). Enhanced mesophilic anaerobic digestion of food waste by thermal pretreatment: Substrate versus digestate heating. *Waste Management*.

[11] Ma J., Duong T. H., Smits M., Verstraete W., Carballa M. (2011). Enhanced biomethanation of kitchen waste by different pre-treatments. *Bioresource Technology*.

[12] Li Y., Jin Y., Li J., Li H., Yu Z., Nie Y. (2017). Effects of thermal pretreatment on degradation kinetics of organics during kitchen waste anaerobic digestion. *Energy*.

[13] Zhang C., Su H., Baeyens J., Tan T. (2014). Reviewing the anaerobic digestion of food waste for biogas production. *Renewable & Sustainable Energy Reviews*.

[14] Mosier N., Hendrickson R., Ho N., Sedlak M., Ladisch M. R. (2005). Optimization of pH controlled liquid hot water pretreatment of corn stover. *Bioresource Technology*.

[15] Liu C., Li H., Zhang Y., Liu C. (2016). Improve biogas production from low-organic-content sludge through high-solids anaerobic co-digestion with food waste. *Bioresource Technology*.

[16] Qiao W., Yan X., Ye J., Sun Y., Wang W., Zhang Z. (2011). Evaluation of biogas production from different biomass wastes with/without hydrothermal pretreatment. *Journal of Renewable Energy*.

[17] Labatut R. A., Angenent L. T., Scott N. R. (2014). Conventional mesophilic vs. thermophilic anaerobic digestion: Atrade-off between performance and stability?. *Water Research*.

[18] Kim M., Ahn Y.-H., Speece R. E. (2002). Comparative process stability and efficiency of anaerobic digestion; mesophilic vs. thermophilic. *Water Research*.

[19] Kastner V., Somitsch W., Schnitzhoger W. (2012). The anaerobic fermentation of FW: a comparison of two bioreactor systems. *J. Clean. Prod*.

[20] Jiang D., Ge X., Zhang Q., Li Y. (2016). Comparison of liquid hot water and alkaline pretreatments of giant reed for improved enzymatic digestibility and biogas energy production. *Bioresource Technology*.

[21] Overend R. P., Chornet E., Gascoigne J. A. (1987). Fractionation of Lignocellulosics by Steam-Aqueous Pretreatments. *Philosophical Transactions of The Royal Society A Mathematical Physical and Engineering Sciences*.

[22] Nguyen D. D., Chang S. W., Jeong S. Y. (2016). Dry thermophilic semi-continuous anaerobic digestion of food waste: Performance evaluation, modified Gompertz model analysis, and energy balance. *Energy Conversion and Management*.

[23] Nielfa A., Cano R., Vinot M., Fernández E., Fdz-Polanco M. (2015). Anaerobic digestion modeling of the main components of organic fraction of municipal solid waste. *Process Safety and Environmental Protection*.

[24] Li Y., Jin Y., Li J., Li H., Yu Z. (2016). Effects of thermal pretreatment on the biomethane yield and hydrolysis rate of kitchen waste. *Applied Energy*.

[25] Donoso-Bravo A., Pérez-Elvira S. I., Fdz-Polanco F. (2010). Application of simplified models for anaerobic biodegradability tests. Evaluation of pre-treatment processes. *Chemical Engineering Journal*.

[26] Achinas S., Euverink G. J. W. (2016). Theoretical analysis of biogas potential prediction from agricultural waste. *Resource-Efficient Technologies*.

[27] APHA. (2005). *Standard Methods for the Examination of Water and Wastewater*.

[28] APHA Standard Methods for the Examination of Water and Wastewater (Methods: 5220 C. Closed Reflux Titrimetric Method), 1998.

[29] Finnish Standard Association (1988). *Determination of chemical oxygen demand (CODCr) in water with closed tube method, oxidation with dichromate. SFS 5504*.

[30] Lowry O. H., Rosebrough N. J., Farr A. L., Randall R. J. (1951). Protein measurement with the Folin phenol reagent. *The Journal of Biological Chemistry*.

[31] Ren J., Manzardo A., Mazzi A., Zuliani F., Scipioni A. (2015). Prioritization of bioethanol production pathways in China based on life cycle sustainability assessment and multicriteria decision-making. *The International Journal of Life Cycle Assessment*.

[32] Mardani A., Zavadskas E. K., Govindan K., Senin A. A., Jusoh A. (2016). VIKOR technique: A systematic review of the state of the art literature on methodologies and applications. *Sustainability *.

[33] Yeshanew M. M., Frunzo L., Lens P. N., Pirozzi F., Esposito G. (2016). Mass Loss Controlled Thermal Pretreatment System to Assess the Effects of Pretreatment Temperature on Organic Matter Solubilization and Methane Yield from Food Waste. *Frontiers in Environmental Science*.

[34] Krishna D., Kalamdhad A. S. (2014). Pre-treatment and anaerobic digestion of food waste for high rate methane production - A review. *Journal of Environmental Chemical Engineering (JECE)*.

[35] Mukamel S. (2000). Multidimensional femtosecond correlation spectroscopies of electronic and vibrational excitations. *Annual Review of Physical Chemistry*.

[36] Hayashi T., Zhuang W., Mukamel S. (2005). Electrostatic DFT map for the complete vibrational amide band of NMA. *The Journal of Physical Chemistry A*.

[37] Koch K., Helmreich B., Drewes J. E. (2015). Co-digestion of food waste in municipal wastewater treatment plants: Effect of different mixtures on methane yield and hydrolysis rate constant. *Applied Energy*.

[38] Esposito G., Frunzo L., Giordano A., Liotta F., Panico A., Pirozzi F. (2012). Anaerobic co-digestion of organic wastes. *Reviews in Environmental Science and Bio/Technology*.

[39] Deepanraj B., Sivasubramanian V., Jayaraj S. (2015). Kinetic study on the effect of temperature on biogas production using a lab scale batch reactor. *Ecotoxicology and Environmental Safety*.

